# A multi-omics analysis reveals that the lysine deacetylase ABHD14B influences glucose metabolism in mammals

**DOI:** 10.1016/j.jbc.2022.102128

**Published:** 2022-06-11

**Authors:** Abinaya Rajendran, Amarendranath Soory, Neha Khandelwal, Girish Ratnaparkhi, Siddhesh S. Kamat

**Affiliations:** Department of Biology, Indian Institute of Science Education and Research (IISER) Pune, Maharashtra, India

**Keywords:** ABHD14B, lysine deacetylase, transcriptomics, metabolomics, glucose metabolism, ABHD14B, α/β-hydrolase domain containing protein # 14B, CoA, coenzyme-A, DEGs, differentially expressed genes, DPBS, Dulbecco’s phosphate-buffered saline, GO, gene ontology, GTT, glucose tolerance test, HAT, histone acetyltransferase, HDAC, histone deacetylase, IDH1, isocitrate dehydrogenase 1, KAT, lysine acetyltransferase, KDAC, lysine deacetylase, PTM, posttranslational modification, PTT, pyruvate tolerance test, PKAc, protein lysine acetylation, TCA, tricarboxylic acid cycle

## Abstract

The sirtuins and histone deacetylases are the best characterized members of the lysine deacetylase (KDAC) enzyme family. Recently, we annotated the “orphan” enzyme ABHD14B (α/β-hydrolase domain containing protein # 14B) as a novel KDAC and showed this enzyme’s ability to transfer an acetyl-group from protein lysine residue(s) to coenzyme-A to yield acetyl-coenzyme-A, thereby, expanding the repertoire of this enzyme family. However, the role of ABHD14B in metabolic processes is not fully elucidated. Here, we investigated the role of this enzyme using mammalian cell knockdowns in a combined transcriptomics and metabolomics analysis. We found from these complementary experiments *in vivo* that the loss of ABHD14B results in significantly altered glucose metabolism, specifically the decreased flux of glucose through glycolysis and the citric acid cycle. Further, we show that depleting hepatic ABHD14B in mice also results in defective systemic glucose metabolism, particularly during fasting. Taken together, our findings illuminate the important metabolic functions that the KDAC ABHD14B plays in mammalian physiology and poses new questions regarding the role of this hitherto cryptic metabolism-regulating enzyme.

Posttranslational modifications (PTMs) are innate cellular mechanisms that tightly regulate many important physiological processes such as chromatin remodeling, transcription, DNA repair, cellular signaling, protein folding, autophagy, apoptosis, and central metabolic pathways (1, 2, 3). While >200 PTMs have been identified to date, only a handful of them have been thoroughly investigated (*e.g.,* phosphorylation, acetylation, and methylation) (1). Protein lysine acetylation (PKAc) is the second most abundant PTM in cells (after phosphorylation) that was identified over half a century ago; yet, its widespread abundance and physiological importance have only been reported recently (4, 5, 6, 7). PKAc was initially thought to be restricted only to histones or the enzymes acetylating them and were therefore monikered histone acetyltransferases (HATs) (8, 9). However, recent studies have shown that numerous nonhistone proteins are also acetylated by this enzyme class (7), and hence, the nomenclature for this enzyme family has been expanded to lysine acetyltransferase (KAT) (Fig. 1*A*) (8). PKAc neutralizes the positive charge on protein lysine residue(s) (*e.g.,* histones, transcription factors and regulators, and nuclear proteins/receptors) alters their protein–protein or protein–DNA interactions and in doing so, is thought to activate transcription by disrupting such interactions (10). PKAc has also been linked to the regulation of metabolism, as recent studies have shown that the activity of key enzymes involved in central metabolism (*e.g.,* glycolysis, gluconeogenesis, tricarboxylic acid cycle (TCA) cycle, urea cycle, fatty acid β-oxidation, and glycogen metabolism) are spatiotemporally regulated and/or contextually modulated by this PTM (6, 7, 11, 12).

**Figure 1:**
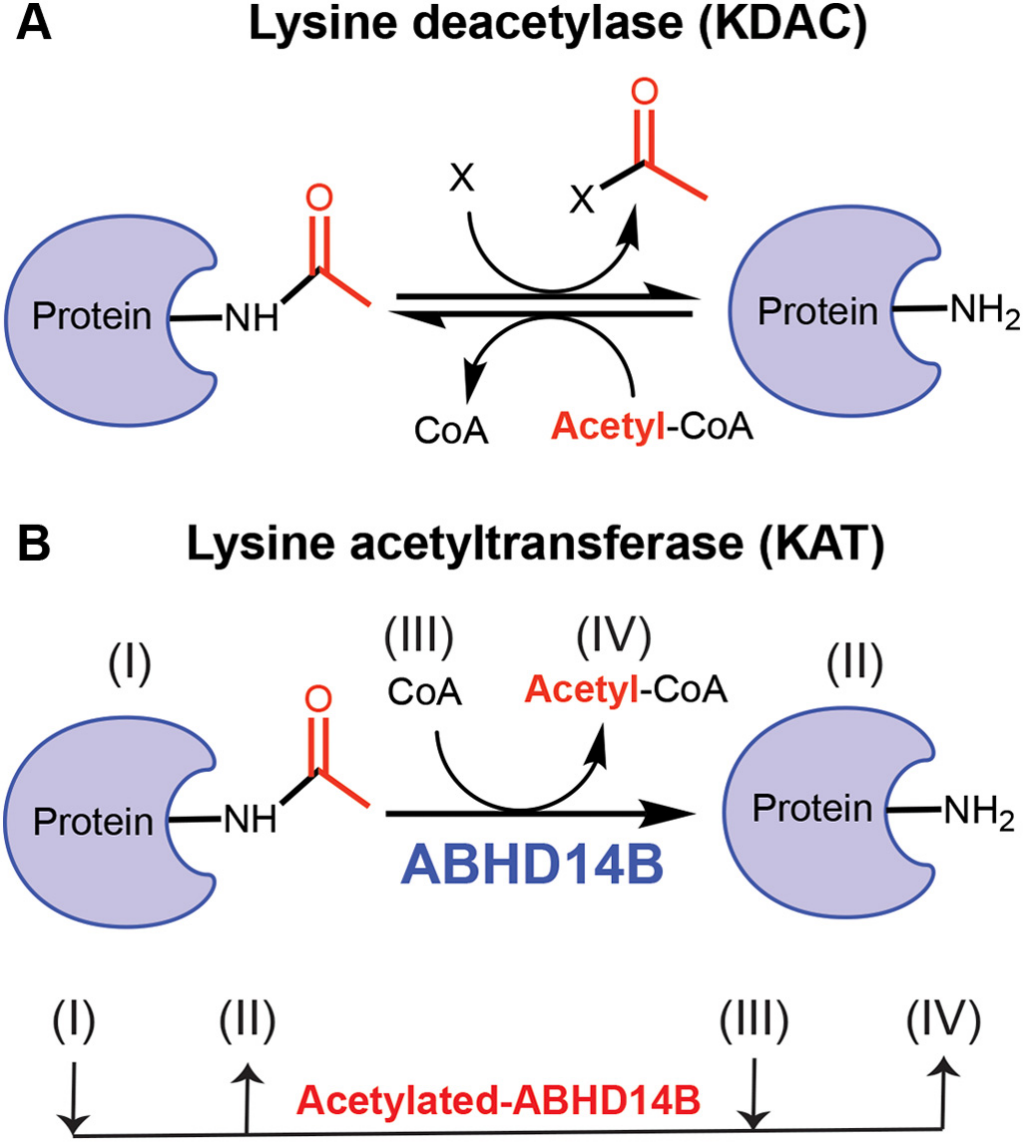
**KATs/KDACs and ABHD14B.***A*, the enzymatic reactions catalyzed by KATs (*e.g.,* HATs) and KDACs (*e.g.,* HDACs, sirtuins, ABHD14B). For the KDAC catalyzed reactions, X = water for HDACs, NAD^+^ for sirtuins, or Co-A for ABHD14B. *B*, the enzymatic reaction catalyzed by the novel KDAC ABHD14B (*top*), using a distinct ping pong mechanism (*below*) conserved for all metabolic serine hydrolase enzymes (24, 28). ABHD14B, α/β-hydrolase domain containing protein # 14B; HATs, histone acetyltransferases; KATs, lysine acetyltransferases; KDACs, lysine deacetylases.

Given its abundance and unlike other PTMs, PKAc is reversible, and the removal of the acetyl group from lysine residue(s) *via* an amide bond cleavage is catalyzed by enzymes known as lysine deacetylases (KDACs) (Fig. 1*A*) (13). Counter to KATs, KDACs deacetylate histones and/or other nuclear proteins remodel the chromatin back into a heterochromatin state and are therefore thought to repress transcription (11, 13, 14). Until recently, KDACs comprised of two enzyme classes: (i) histone deacetylases (HDACs) (moniker, given their ability to deacetylate histones) (13, 14, 15) and (ii) sirtuins (16, 17, 18, 19). The catalytic mechanisms of both these enzyme classes and the eventual fate of the acetyl group are quite distinct. The HDACs for example use a divalent metal cation (generally Zn^2+^) to activate a nucleophilic water molecule that hydrolyses the amide bond of an acetylated lysine residue to give free acetate as the product (13, 14, 15). The sirtuins on the other hand are NAD^+^ dependent and yield nicotinamide and 2’/3′-*O*-acetyl-ADP-ribose as the end products of the KDAC reaction (16). Like KATs, KDACs also regulate important physiological processes like cell growth and differentiation, energy production, glucose and lipid metabolism, longevity, and organelle biogenesis (4, 11). Given their physiological importance, deregulation in the cellular balance of KAT/KDAC activity has been linked to numerous human metabolic pathophysiological conditions like diabetes, obesity, cancer, neurodegenerative disorders, cardiac hypertrophy, and autoimmunity (20, 21, 22, 23).

Recently, we annotated the orphan enzyme α/β-hydrolase domain containing protein # 14B (ABHD14B) as a novel KDAC, thus expanding this enzyme family’s repertoire (24). Nearly 2 decades ago, the three-dimensional structure of human ABHD14B was solved, and a yeast two-hybrid screen identified this enzyme as an interactor of the HAT domain of the transcription factor TAF_II_250 (25). The reported structural studies showed that ABHD14B possessed the canonical ABHD fold (25, 26, 27), an invariant catalytic triad (Ser-His-Asp), and based on its protein sequence, was subsequently categorized as an outlying member of the metabolic serine hydrolase family (28). We recently showed that ABHD14B was able to transfer an acetyl group from posttranslationally modified protein lysine residue to coenzyme-A (CoA), thus making the biologically important acetyl-CoA (Fig. 1*B*) (24). Genetic knockdown of ABHD14B in mammalian cells showed significantly increased cellular PKAc and decreased cellular concentrations of acetyl-CoA, thus confirming our *in vitro* functional annotation to more physiological cellular setting (24). What distinguishes ABHD14B from the HDACs and sirtuins are (i) the use of CoA as a co-substrate and the end fate of the acetyl group (formation of acetyl-CoA) and (ii) the catalytically conserved ping-pong mechanism conserved for all metabolic serine hydrolase enzymes (Fig. 1*B*) (24).

To investigate the physiological role of ABHD14B, we developed an exquisitely selective antibody against mammalian ABHD14B (24) and surveyed the tissue distribution of ABHD14B in mice. Quite interestingly, this protein profiling study showed that ABHD14B had restricted expression only in the metabolically active tissues (liver and kidneys) (24), leading us to hypothesize that this enzyme activity might be playing an important function in regulating metabolism and in turn cellular energetics. Following up on this study, here, we report an integrated, yet very complementary, transcriptomics, and metabolomics analysis in mammalian HEK293T cells, where ABHD14B expression is knocked down. These experiments together suggest that ABHD14B plays an important role in modulating glucose metabolism in cells and in turn also regulates cellular energetics. Further, from studies in mice, we find that disruption of hepatic ABHD14B especially during fasting, disturbs homeostatic systemic glucose metabolism, and in doing so, significantly alters the organismal energetic status. Taken together, our findings collectively establish for the first time, a link between (hepatic) ABHD14B and glucose metabolism in mammals.

## Results

### Transcriptomics shows ABHD14B regulates central metabolic pathways

**Figure 2:**
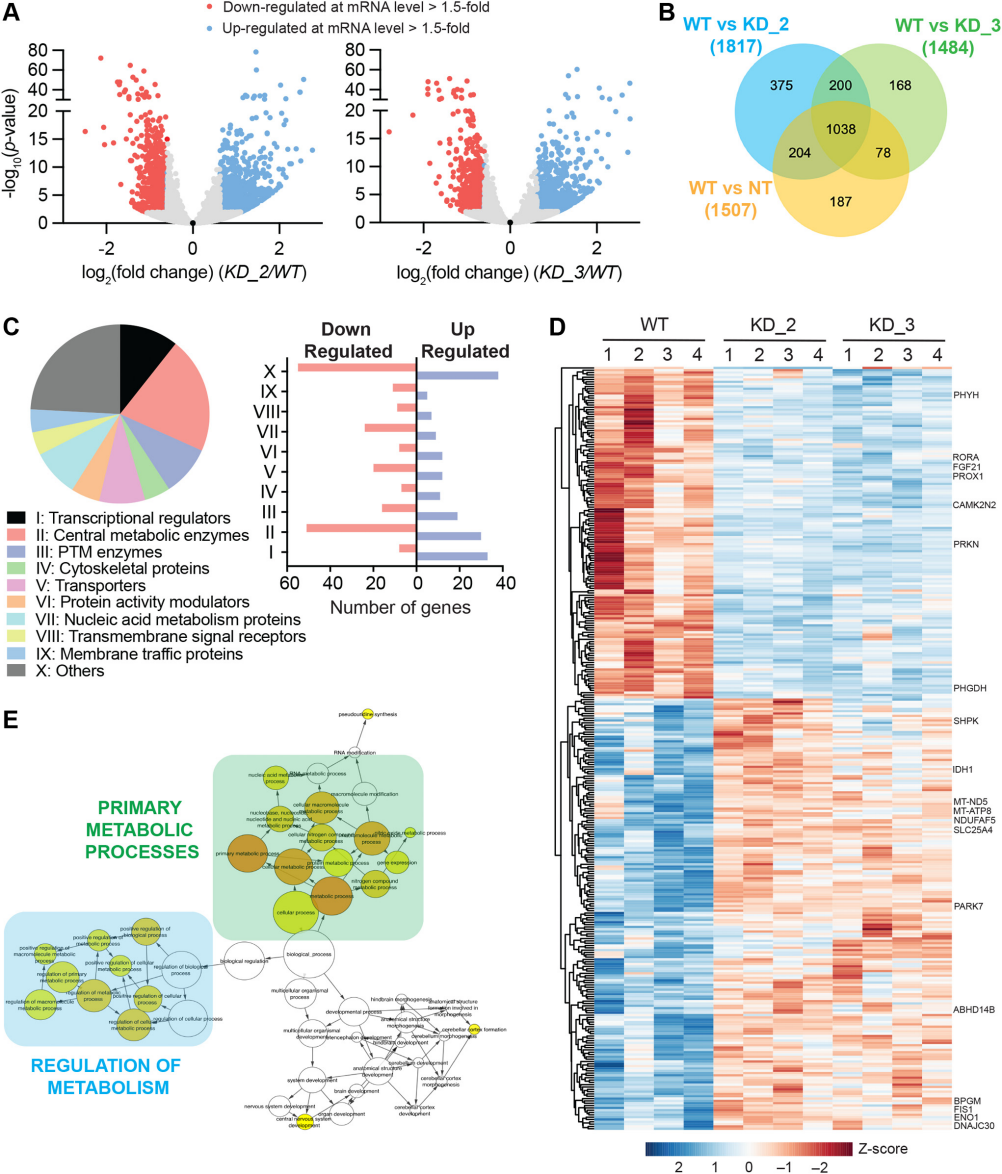
**ABHD14B knockdown causes transcriptomics changes in HEK293T cells**. *A*, volcano plot showing differentially expressed genes (DEGs) in KD_2 and KD_3 HEK293T cells relative to WT HEK293T cells as determined by RNA-sequencing. The data represents mean values from four independent biological experiments. A cut-off of adjusted *p*-value < 0.01 was set for the genes, with a change of > 1.5-fold. Based on this filter, upregulated and downregulated genes are colored in *blue* and *red*, respectively. *B*, Venn diagram analysis of the DEGs from the transcriptomics analysis of KD_2, KD_3, and NT HEK293T cells relative to WT HEK293T cells used to eliminate the off-targets of NT HEK293T cells from subsequent bioinformatics analysis. *C*, gene ontology annotation of the DEGs showing various functional protein classes (*left*) and the number of DEGs from each functional protein class (*right*) from the KD_2 and/or KD_3 HEK293T cells relative to WT HEK293T cells using the PANTHER classification system (29). *D*, hierarchical clustering analysis of the various DEGs involved in metabolic pathways and processes as per the MGI database (30), showing like-wise (similar trend) upregulation or downregulation in expression profiles in KD_2 and/or KD_3 HEK293T cells relative to WT HEK293T cells. The gene names written on the graph were considered important metabolic genes as part of this study, showing stark changes in KD_2 and/or KD_3 HEK293T cells relative to WT HEK293T cells, and are further summarized in Figure 5. *E*, a cellular network analysis using the Cytoscape program (31), showing that, amongst the various DEGs identified from the MGI database, primary metabolic processes (*e.g.,* glycolysis and TCA cycle) and regulators of these processes are the most enriched or overrepresented biological pathways from this bioinformatics analysis. For (*A-E*), complete information of all the DEGs from all these analyses are available in Table S1. TCA, tricarboxylic acid cycle.

Preliminary studies have shown that by its putative interactions with the HAT domain of TAF_II_250, ABHD14B can regulate transcriptional activity in mammalian cells (25). We recently annotated ABHD14B as a novel KDAC, and other KDACs (*e.g.,* sirtuins, HDACs) are known regulators of transcription and in turn metabolic activity in mammals. Given this precedence, we decided to perform a transcriptomics analysis in mammalian cells, where ABHD14B was depleted. We have previously shown that plasmids KD_2 and KD_3 produced robust and significant (>90%) knockdown of ABHD14B in mammalian HEK293T cells relative to a control nontargeting (NT) plasmid and chose this system for our transcriptomics studies (Fig. S1*A*) (24). In this study, we also included untreated “wild-type” (WT) HEK293T cells as an additional control to negate any “off” targets of the control NT plasmid and enrich transcriptional changes specifically brought about specifically by the depletion of ABHD14B in this cell line. Using standard protocols, RNA was isolated from all these HEK293T cell lines (*i*.*e.*, KD_2, KD_3, NT, and WT), and transcriptome analysis was performed using the Ilumina NextSeq platform as per manufacturer’s instructions (Fig. S1*B*). First, to ensure relatedness between samples of a particular HEK293T cell line (*i*.*e.*, KD_2, KD_3, NT, and WT), after examining the raw reads (Fig. S1*C*), we performed a principal component analysis amongst all these samples (Fig. S1*D*) and found tight clustering of samples of a particular cell line, suggesting that a particular HEK293T cell line (*i*.*e.*, KD_2, KD_3, NT, and WT) behaved like wise in the transcriptomics analysis. Next, we looked for the differentially expressed genes (DEGs) in the KD_2 and KD_3 HEK293T cell lines relative to WT HEK293T cells and found 1817 (1017 upregulated and 800 downregulated) and 1484 (841 upregulated and 643 downregulated) DEGs respectively that passed the set threshold (>1.5-fold change and adjusted *p*-value < 0.01) from this analysis (Fig. 2*A* and Table S1). Here, we also found that relative to WT HEK293T cells, NT-treated HEK293T cells also showed 1507 (832 upregulated and 675 downregulated) DEGs (Fig. S1*E* and Table S1), and therefore, we decided to remove such DEGs (off-targets of NT) from further analysis. For this, we assessed the DEGs data from all the HEK293T cell lines using a Venn diagram (Fig. 2*B*) and found a total of 743 genes (Table S1) that were differentially enriched (410 upregulated and 333 downregulated) in either KD_2 (375 genes, 205 upregulated and 170 downregulated) or KD_3 (168 genes, 91 upregulated and 77 downregulated) or both (200 genes, 114 upregulated and 86 downregulated) HEK293T cell line.

Having shortlisted 743 putative DEGs from the aforementioned transcriptome analysis, leveraging publicly available (open-source) bioinformatics platforms, next, we wanted to identify the possible biological pathways that might be altered as a result of depleting the KDAC ABHD14B in HEK293T cells. Towards this, we first decided to perform a gene ontology (GO) survey and searched these 743 DEGs in the PANTHER classification system (http://www.pantherdb.org) (29). From this analysis, we found that out of the 743 DEGs, 369 were annotated in the PANTHER classification database, while the remaining 374 DEGs were classified (or listed) as uncharacterized or unknown in this classification system (Table S1). Amongst the 369 DEGs annotated in PANTHER, we found that enzymes involved in central metabolic processes (81 genes, 30 upregulated and 51 downregulated) and transcriptional regulators modulating metabolic processes (41 genes, 33 upregulated and eight downregulated) were the most enriched class of genes (Fig. 2*C*). Several other proteins (or enzymes) involved in metabolic processes such as PTM modulating enzymes, protein activity modulators, and enzymes regulating nucleic acid metabolism (Fig. 2*C* and Table S1) were also represented in this GO study and suggested that ABHD14B might have a function in the regulation of metabolism.

To avoid any database bias, we performed a similar GO analysis in the MGI database (http://www.informatics.jax.org) (30) and found that of the 743 DEGs, a significant majority (329 genes, 178 upregulated and 151 downregulated) had putative roles to play in central metabolic processes and/or its regulation, especially glucose metabolism (Table S1). We performed a hierarchical clustering analysis of these 329 DEGs involved in metabolism from the GO search from the MGI database for all the replicates from the transcriptome analysis and found a significant change in their expression profiles in almost all the KD_2 or KD_3 HEK293T cell replicates relative to WT HEK293T cell replicates (Fig. 2*D*). Further, using Cytoscape (https://cytoscape.org) (31), we performed a biological network analysis on these 329 DEGs putatively involved in metabolism and found that cellular or primary metabolic processes (*e.g.,* glycolysis, citric acid cycle) were the most overrepresented pathway annotation, followed by regulation of metabolic processes (Fig. 2*E* and Table S1). Finally, to validate the findings from the transcriptomics analysis, we performed a real-time qPCR experiment on a handful of DEGs and found from this experiment that these DEGs showed changes and trends similar to those observed from the transcriptomics analysis (Fig. S1*F*). Taken together, the transcriptomics study coupled with the GO and bioinformatics analysis from PANTHER and MGI database strongly suggest that ABHD14B regulates expression of numerous proteins, particularly enzymes involved in central metabolic processes, especially glucose metabolism and/or transcriptional regulators of primary metabolic processes.

### Metabolomics shows ABHD14B regulates glucose metabolism

**Figure 3:**
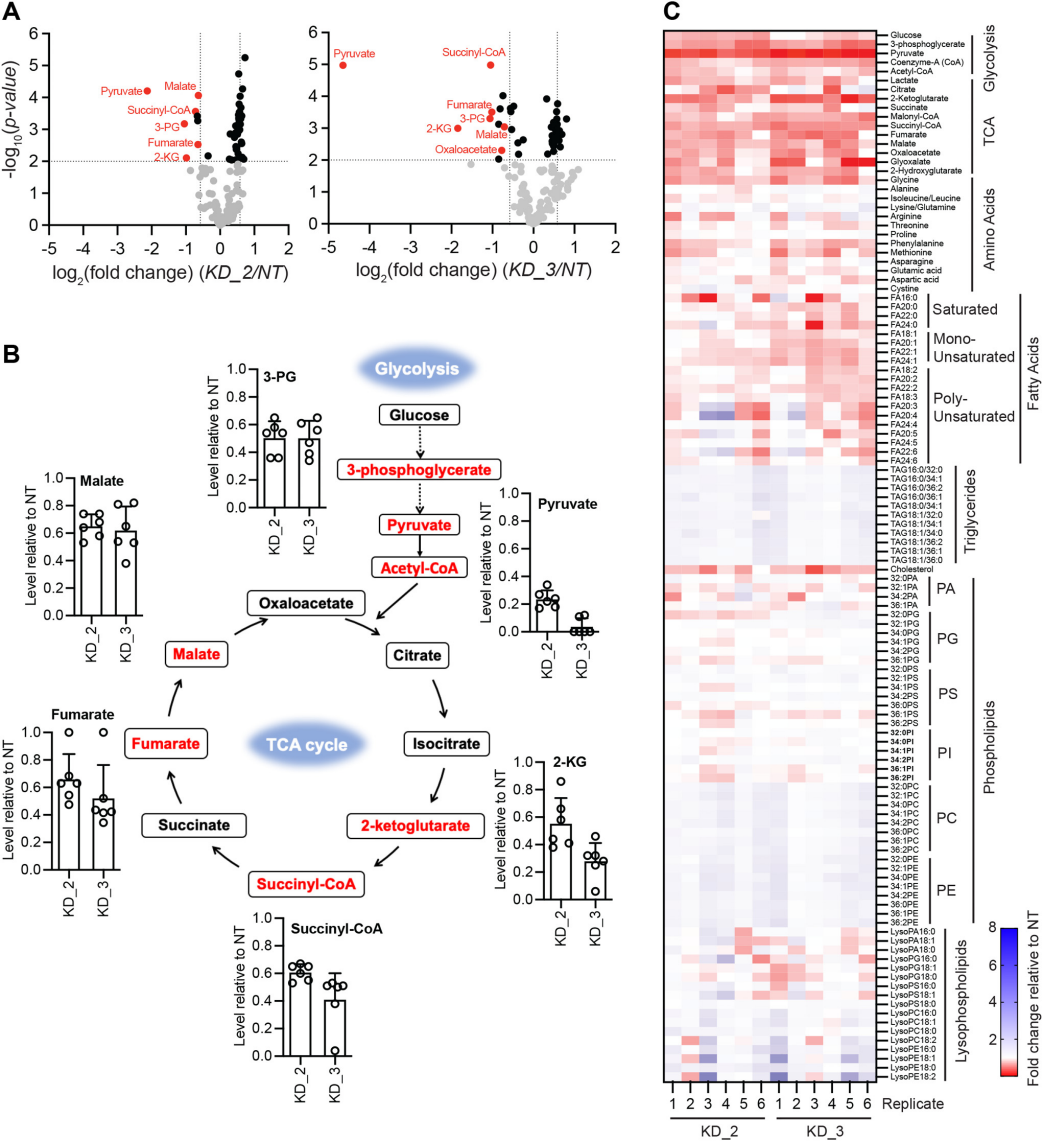
**ABHD14B knockdown causes altered glucose metabolism in HEK293T cells.***A*, volcano plot showing differentially changing metabolites in KD_2 and KD_3 HEK293T cells relative to NT HEK293T cells (all values normalized to WT HEK293T cells) as determined by established LC-MS/MS analysis. The data represent mean values from six independent biological experiments, with a cut-off of adjusted *p*-value < 0.01 (*dashed line* parallel to x-axis) and a > 1.5-fold change (*dashed lines* parallel to y-axis). Based on this filter, the metabolites or intermediates involved in central glucose metabolic pathways that have reduced cellular concentrations are colored in *red*. *B*, relative quantification of intermediates of the glycolysis and TCA cycle, showing significantly reduced cellular concentrations in KD_2 and KD_3 HEK293T cells relative to NT HEK293T cells (all values normalized to WT HEK293T cells). Bars represents mean ± standard deviation from six biological replicates (independent experiments) per group. *C*, heat map plot from every individual experiment from KD_2 and KD_3 HEK293T cells relative to NT HEK293T cells (all values normalized to WT HEK293T cells), showing relative cellular concentrations of metabolites and/or intermediates from different central metabolic pathways (glycolysis, TCA cycle) and for various type of biomolecules (*e.g.,* sugars, amino acids, lipids). Colors in *red* and *blue* represent a decrease and increase in cellular concentration respectively. For (*A–C*), complete information of all the metabolites assessed in this study along with their quantification are available in Table S2. TCA, tricarboxylic acid cycle.

Since the transcriptomics analysis hinted at a role for ABHD14B in regulating cellular central metabolic pathways, we decided to perform a focused metabolomics analysis and determine if any metabolites and/or central pathways were altered in mammalian cells following ABHD14B depletion. Toward this, using established metabolite extraction and LC-MS/MS methods (Fig. S2*A* and Table S2) (32, 33, 34, 35), we performed semi-quantitative untargeted (information-dependent acquisition) and/or targeted (multiple reaction monitoring) metabolomics analysis in KD_2 and KD_3 HEK293T cells, where ABHD14B was significantly knocked down relative to NT control. Like the transcriptomics analysis, in the metabolomics studies, untreated WT HEK293T cells were also used as an additional control, and metabolites analyzed from all treated cell lines (NT, KD_2, and KD_3) were normalized to WT HEK293T cells to ensure any “off” target effects were nullified in this metabolomics analysis. From this LC-MS/MS–based metabolomics analysis, the metabolites were considered as “hits” (significantly altered) if they passed the selection filter, *i.e.,* a >1.5-fold change (increase or decrease) with a stringent *p*-value < 0.01, in both KD_2 and KD_3 groups relative to the NT control group. While we were able to confidently identify and semi-quantitate > 200 unique metabolites, only a few (∼30) passed the aforementioned selection filter and evaluated further (Fig. 3*A*). Corroborating the transcriptomics data, prominent among the altered metabolites, interestingly, were several key intermediates from the glycolysis pathway and TCA cycle (or citric acid cycle) that were significantly reduced in their cellular concentrations upon ABHD14B depletion in HEK293T cells (Fig. 3, *A* and *B* and Table S2). Among all the altered metabolites, the end intermediate and/or product of glycolysis, pyruvate, was the most substantially reduced in its cellular concentrations in both KD_2 (∼85%) and KD_3 (∼95%) groups (Fig. 3*B*), while another high energy glycolytic intermediate, 3-phosphoglycerate (3-PG), was also significantly reduced by ∼50% in both KD_2 and KD_3 HEK293T cells (Fig. 3*B*). Further, we also found from this metabolomics analysis that downstream of glycolysis, several important TCA cycle intermediates namely, 2-ketoglutarate (2-KG) (or α-ketoglutarate), succinyl-CoA, fumarate, and malate were also consistently reduced in their cellular concentrations by 40 to 60% in both KD_2 and KD_3 HEK293T cells (Fig. 3*B*).

Since the transcriptomics analysis suggested that ABHD14B was possibly involved in regulating expression of genes involved in central or primary metabolic pathways, we also looked in more detail at various other metabolites (*e.g.,* amino acids, fatty acids, neutral and phospholipids) (Fig. 3*C* and Table S2). Here, we found that relative to most of the other central metabolic pathways, upon depletion of ABHD14B in HEK293T cells, glycolysis and TCA cycle were by far the most perturbed central (or primary) metabolic pathways, in that most metabolites of these pathways were significantly reduced in cellular concentrations. Interestingly, apart from the aforementioned metabolites (Fig. 3*B* and Table S2), we found that a few more intermediates on glycolysis and/or TCA cycle namely Co-A/acetyl-CoA and oxaloacetate, along with some shunt and/or byproducts of these pathways (glyoxylate, lactate, and 2-hydroxyglutarate), all showed a consistent reduction (∼20–40%) in cellular concentration upon ABHD14B knock down, but did not pass the strict selection filters described earlier (Figs. 3*C*, S2*B* and Table S2). Besides glycolysis and the TCA cycle, we did not find any altered cellular levels for various amino acids, free fatty acids (saturated, monounsaturated or polyunsaturated), most phospholipids (phosphatidic acid, phosphatidylglycerol, phosphatidylserine, and phosphatidylinositol), and all measured lysophospholipids as a result of ABHD14B depletion in HEK293T cells (Fig. 3*C* and Table S2). We did however notice that there was a modest increase (∼30–40%) in cellular triglycerides and abundant phospholipids (phosphatidyl-cholines and -ethanolamines) levels with a concomitant decrease in cellular cholesterol levels in HEK293T cells with depleted ABHD14B levels (Figs. 3*C*, S2*B* and Table S2). Finally, to validate the metabolomics data, we performed a complementary lactate production assay in all the HEK293T cell lines and found that the depletion of ABHD14B in HEK293T cells indeed resulted in depletion of cellular lactate levels, with a concomitant increase in the secreted lactate concentrations (∼2-fold) (Fig. S2*C*). This finding further supports that depletion of ABHD14B alters the flux of glucose metabolism through both the glycolysis and TCA cycles, by shunting pyruvate to produce lactate that is eventually secreted from cells, thus resulting in decreased cellular levels of both pyruvate and lactate.

### Hepatic ABHD14B regulates systemic glucose homeostasis

**Figure 4:**
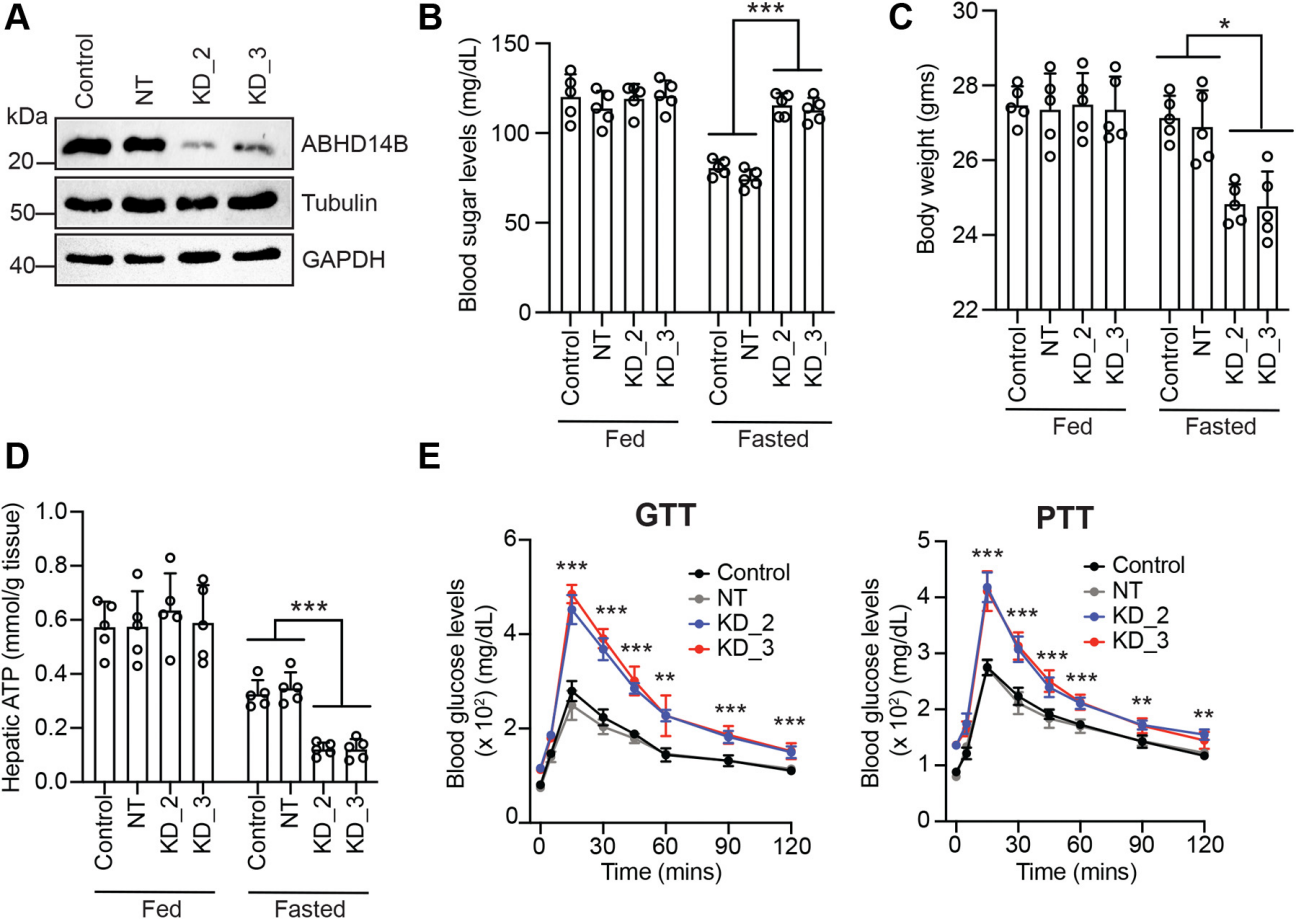
**Hepatic ABHD14B regulates glucose metabolism in mice**. *A*, representative Western blots confirming the knockdown of ABHD14B at a protein level (>90%) in mice liver following transfections with KD_2 or KD_3 plasmids, relative to a non-targeting (NT) plasmid or no plasmid “control” 48 h posttransfection. α-Tubulin and GAPDH were used as protein loading controls for this immunoblotting experiment. This Western blot analysis was performed three times with reproducible results each time. *B*–*D*, blood glucose concentrations (*B*), body weights (*C*), and hepatic ATP concentrations (*D*) of mice from all experimental groups (control, NT, KD_2 and KD_3) after 6 h fasting. *E*, blood glucose concentrations in mice from all experimental groups (control, NT, KD_2, and KD_3) during the course of a glucose tolerance test (GTT) or a pyruvate tolerance test (PTT) performed post 6 h of fasting. Data for (*B-E*) represent mean ± standard deviation from five biological replicates (independent experiments) per group. ∗*p* < 0.05, ∗∗*p* < 0.01, and ∗∗∗*p* < 0.001 for KD_2 or KD_3 group *versus* control or NT group by Student’s two-tailed unpaired parametric *t* test. ABHD14B, α/β-hydrolase domain containing protein # 14B.

Our protein profiling studies in mice showed that ABHD14B is exclusively expressed in metabolically active tissues (*i.e*., liver and kidneys), and therefore, we wanted to investigate, the role that hepatic ABHD14B plays in systemic metabolism. To the best of our knowledge, there are no pharmacological tools (*e.g.,* ABHD14B specific inhibitors) and/or genetic animal models (*e.g.,* ABHD14B knockout mice) to study the *in vivo* functions of ABHD14B, and therefore, we used an established nonviral *in vivo* transfection strategy to knockdown hepatic ABHD14B in mice (36, 37, 38) using the KD_2 and KD_3 plasmids that have produced robust knockdown of ABHD14B (>95%) in mammalian cells (Fig. S3*A*) (24). In this experimental paradigm, 48 h posttransfection, we found by immunoblotting that ABHD14B was substantially depleted (>90%) in the soluble liver proteomes of mice that were administered with KD_2 or KD_3 plasmids, relative to the NT control or no plasmid (control) (Fig. 4*A*). Taking cues from the transcriptomics and metabolomics study from mammalian cells, we postulated that hepatic ABHD14B might have a regulatory function in glucose metabolism, and hence, we investigated the effects of ABHD14B depletion during a fasting paradigm (Fig. S3*A*). We found that upon 6 h of fasting, mice from the control and NT groups had significantly reduced blood glucose levels, while interestingly, mice from the KD_2 and KD_3 groups did not show any reduction in blood glucose levels (Fig. 4*B*). During the course of this experiment, we noticed by visual inspection that upon 6 h of fasting, mice from the KD_2 or KD_3 group, but not from the control or NT group, appeared very sluggish and lost significant body weight during fasting (∼15%) (Fig. 4*C*). Given this observation, we decided to measure hepatic adenosine 5′-triphosphate (ATP) levels of all mice, as this is often a measure of organismal energy status (39, 40, 41). Here, we found that during fed conditions, mice from all experimental groups had comparable hepatic ATP levels, while upon 6 h of fasting, mice from all experimental groups had decreased concentrations of hepatic ATP (Fig. 4*D*). However, the difference between fed-fasted hepatic ATP levels was the most profound for mice from the KD_2 and KD_3 groups compared to the control (or NT) group (Fig. 4*D*).

Following up on this interesting finding, we performed a glucose tolerance test (GTT) and a pyruvate tolerance test (PTT) on mice from all groups post 6 h of fasting using established protocols (Fig. 4*E*) (36, 42) and found that mice from the KD_2 and KD_3 groups had significantly more blood glucose levels in both the GTT and PTT compared to mice from the control group (Fig. 4*E*), while the blood glucose profile for mice from the NT group was comparable to the control group (Fig. 4*E*). The time course profiles from both these tests showed that the differences in blood glucose levels between the mice from the KD_2 or KD_3 group relative to the control (or NT) group were most pronounced at earlier time points (15 or 30 min) (Fig. 4*E*). We confirmed by immunoblotting that at the end of both the GTT and PTT, hepatic ABHD14B levels were still significantly depleted in liver soluble lysates of mice from KD_2 or KD_3 group relative to mice from the NT group (Fig. S3*B*). Besides the liver, the commercially available transfection reagent used by us here, is sometimes known to target knockdown of the same protein in the kidney as well, and to check if this was the case in our experiments, we decided to look at the levels of ABHD14B in the kidneys by immunoblotting. We found no change in ABHD14B levels in the kidney during the course of this experiment (Fig. S3*B*), suggesting that the effects on systemic glucose metabolism were largely, if not exclusively, driven by hepatic ABHD14B. Finally, we also measured serum insulin and glucagon concentrations, as these hormones in mammals, including humans, intricately regulate physiological glucose concentrations during a fed-fasted response (43, 44, 45). Consistent with a fasting response, we found that serum insulin levels decreased, while serum glucagon concentrations increased for all experimental groups (Fig. S3*C*). Further, we found that depleting hepatic ABHD14B had no effect on serum insulin levels between the various experimental groups (Fig. S3*C*). However, upon fasting, we found that serum glucagon levels significantly increased (∼2-fold) for mice from the KD_2 and KD_3 experimental groups compared to the NT or control group (Fig. S3*C*).

## Discussion

PKAc is an abundant and important PTM that regulates many facets of mammalian physiology, and the enzymes that control the formation or degradation of this reversible PTM serve as critical metabolic lynchpins (4, 11). We recently annotated the orphan serine hydrolase enzyme ABHD14B as a KDAC and, in doing so, have expanded the catalytic mechanisms used by this enzyme family for PKAc transferase reactions (24) (Fig. 1). Following up on this discovery, we wanted to map the biological pathways that ABHD14B regulates and/or influences, given its restricted expression in metabolically active tissues. Toward this, we performed an integrated transcriptomics (Fig. 2) and metabolomics (Fig. 3) analysis in HEK293T cells, where ABHD14B was significantly depleted, and found several metabolic pathways, especially those associated with glucose metabolism were significantly altered. To further validate this phenotype in an animal model, in the absence of any pharmacological and/or genetic tools to study ABHD14B function *in vivo*, we successfully used a transient transfection methodology to knockdown ABHD14B specifically in mice liver (Fig. 4). Consistent with studies from HEK293T cells, here, upon ABHD14B depletion in the liver, we found that fasted mice had unchanged blood glucose concentrations, massively elevated glucose levels during a GTT or PTT, and diminished hepatic ATP levels, all together suggestive of a dysregulated glucose metabolic phenotype and possibly an increased anabolic response from hampered glycolysis and TCA cycle flux coupled to a heightened gluconeogenesis response.

**Figure 5:**
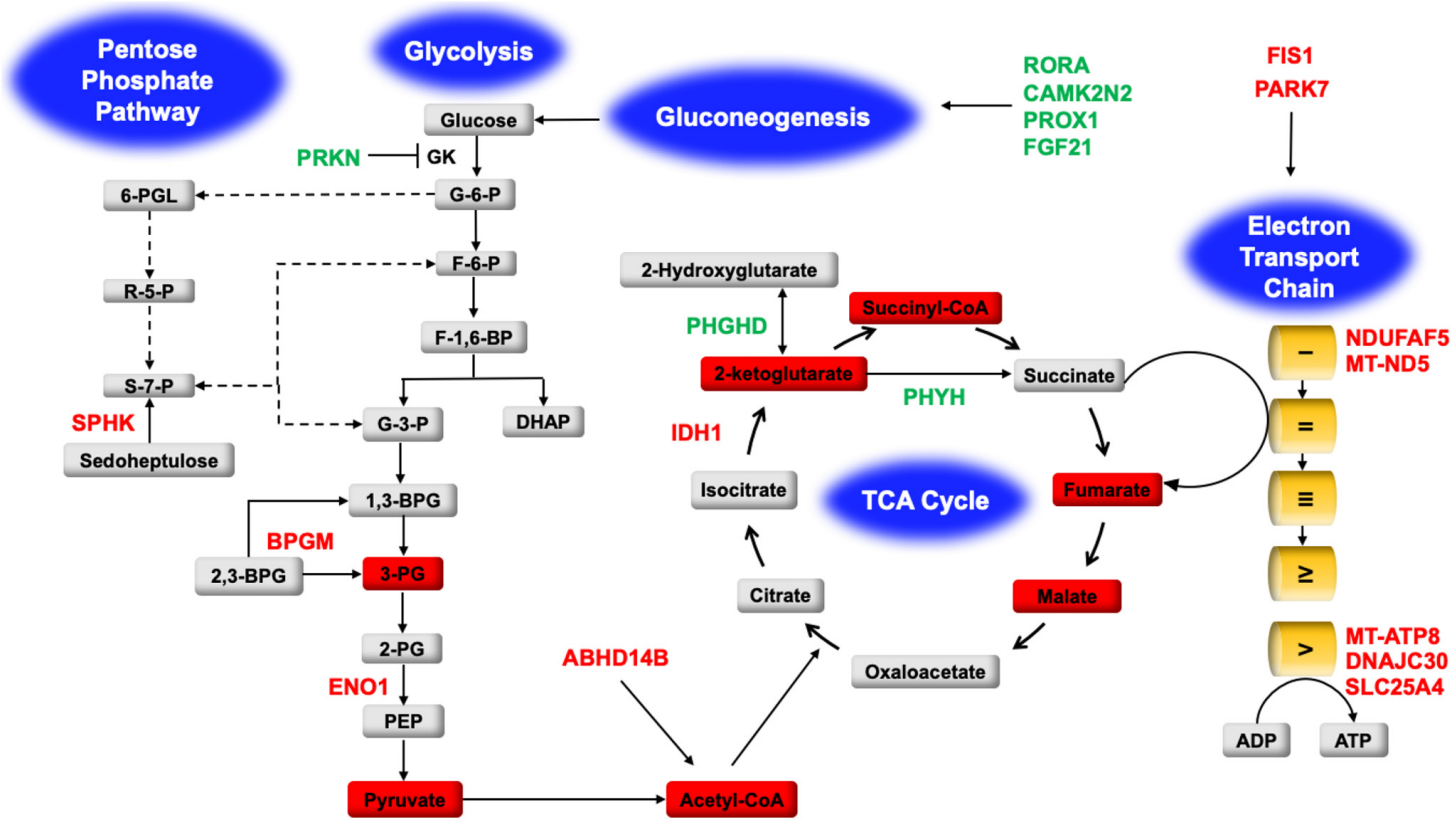
**A model summarizing the transcriptomic and metabolite changes following ABHD14B depletion**. All genes colored in *green* and *red* represent upregulated and downregulated DEGs respectively based on the RNA-sequencing–based transcriptomics analysis performed in HEK293T cells following the knockdown of ABHD14B. The metabolites in *red boxes* represent metabolites significantly reduced in cellular concentrations based on the LC-MS/MS–based metabolomics experiments in HEK293T cells following depletion of ABHD14B. Abbreviations for all genes and metabolites shown in this figure are described in the Discussion section. ABHD14B, α/β-hydrolase domain containing protein # 14B; DEG, differentially expressed genes; TCA, tricarboxylic acid cycle.

Based on the aforementioned data, we propose a model (albeit preliminary) (Fig. 5), summarizing all the transcriptome and metabolite changes associated with dysregulated glucose metabolism. On the glycolysis pathway, we find from our transcriptome analysis, that the expression of Parkin (PRKN) is significantly upregulated, and this E3 ubiquitin ligase is a negative regulator of glucokinase (GK), the enzyme catalyzing the first committed step of glycolysis, where glucose is converted to glucose-6-phosphate (G-6-P) (46, 47, 48). On the same pathway, we also find that the expression of the enzymes bisphosphoglycerate mutase (BPGM) and enolase 1 (ENO1) is significantly downregulated following the depletion of ABHD14B in HEK293T cells. BPGM is responsible for the production of 3-PG during glycolysis, while ENO1 catalyzes the formation of the high energy and short-lived phosphoenolpyruvate (PEP) intermediate from 2-PG, that subsequently drives an energetically downhill reaction to form pyruvate, the end product of glycolysis (49, 50, 51). Our metabolomics studies show a strong correlation to the transcriptomic changes of both BPGM and ENO1; in that, we find that the cellular levels of both 3-PG and pyruvate are substantially diminished (Fig. 3), suggesting that together with the regulatory effects of PKRN, glycolysis is a significantly downregulated pathway in ABHD14B-depleted HEK293T cells. Following glycolysis, its end product pyruvate is converted to acetyl-CoA by the pyruvate dehydrogenase enzyme complex, and acetyl-CoA then feeds into the TCA cycle, thus linking these two-important primary central metabolic processes (47, 50, 51). Previous studies by us have shown that cellular acetyl-CoA is also generated by the KDAC reaction of ABHD14B, and depleting ABHD14B in HEK293T cells results in its decreased cellular levels of acetyl-CoA (24). Since both the cellular pyruvate levels and ABHD14B are substantially reduced, the overall cellular concentrations of acetyl-CoA also concomitantly decrease, which in turn also affects the TCA cycle. Additionally, significantly more lactate is secreted from HEK293T cells upon ABHD14B knockdown, further depleting pyruvate and acetyl-CoA production, presumably from this shunt away from glycolysis and the TCA cycle.

On the TCA cycle (50), we found that the cytosolic isocitrate dehydrogenase 1 (IDH1) was significantly downregulated, and metabolomics confirmed that the cellular concentrations of 2-KG (Fig. 3), the product of the IDH1 catalyzed conversion of isocitrate, were also significantly lowered. Since IDH1 catalyzes the first committed step in the TCA cycle, several other downstream TCA intermediates (succinyl-CoA, fumarate, and malate) also had significantly reduced cellular concentrations (Fig. 3). Interestingly, in peroxisomes, 2-KG is a co-substrate for the conversion of phytanic acid to 2-hydroxy-phytanic acid by the enzyme phytanoyl-CoA 2-hydroxylase (PHYH), so that phytanic acid can be metabolized *via* the β-oxidation pathway (52). We find that on ABHD14B depletion in HEK293T cells, the expression of PHYH is upregulated, and therefore, cellular 2-KG is likely utilized *via* this pathway and thus shunted away from the TCA cycle. Upon ABHD14B depletion, we also found that an upregulation in the expression of the enzyme D-3-phosphoglycerate dehydrogenase (PHGHD), that converts 2-KG to 2-hydroxyglutarate, and this reaction also possibly shunts 2-KG away from the TCA cycle, causing its cellular levels to be lowered (53). Further, we found that the enzyme sedohepulokinase, which phosphorylates sedoheptulose to sedoheptulose-7-phosphate (S-7-P), was downregulated upon ABHD14B depletion. S-7-P directly feeds into the pentose phosphate pathway and eventually gets converted to fructose-6-phosphate and glyceraldehyde-3-phosphate (54, 55), both of which, eventually feed into glycolysis, and by downregulating sedohepulokinase, the contribution of this pathway to sustain glycolysis also seems to be diminished.

Both glycolysis and the TCA cycle are energy generating (catabolic) cellular processes during glucose metabolism, and we find that upon ABHD14B depletion, the cellular concentrations of several intermediates on these pathways are substantially reduced. Not surprisingly, downstream of these pathways, possibly by some as of yet unknown regulatory (anticipatory) mechanism, we find that different components of the electron transport chain protein complex (*e.g.,* NDUFAF5 and MT-ND5 from complex I, and MT-ATP8 and DNAJC30 from complex V) and regulators (activators) of the electron transport chain (*e.g.,* SLC25A4, FIS1, PARK7) that mediate the import of ADP into the mitochondrial matrix for ATP synthesis (56, 57, 58) have lowered expression in HEK293T cells upon ABHD14B depletion. Concomitantly, we also find that several genes (*e.g*., *RORA, CAMK2N2, PROX1*, and *FGF21*) that modulate gluconeogenesis (43, 44, 45, 59, 60, 61, 62, 63) are also upregulated in ABHD14B deplete HEK293T cells. Counter to glycolysis and the TCA cycle, gluconeogenesis is an energy consuming (anabolic) process, and while, as a function of ABHD14B in HEK293T cells, we do not have any direct evidence of heightened gluconeogenesis, consistent with the transcriptome data, our studies in mice do strongly suggest an increased systemic gluconeogenesis phenotype as a function of hepatic ABHD14B. In support of this heightened systemic anabolic response, we find that depleting hepatic ABHD14B results in massively elevated blood glucose levels during a PTT, with a significant decrease in hepatic ATP concentration and net body weight while fasting (Fig. 4). Taken together, our transcriptome and metabolomics data along with the studies in mice provides compelling experimental support in establishing a role for ABHD14B in regulating glucose metabolism and in turn cellular/organismal energy status and suggests that depleting this KDAC hampers (downregulates) both the glycolysis and TCA cycle and presumably upregulates a systemic gluconeogenic response (43, 44, 45, 59, 60, 61, 62, 63).

**Figure 6:**
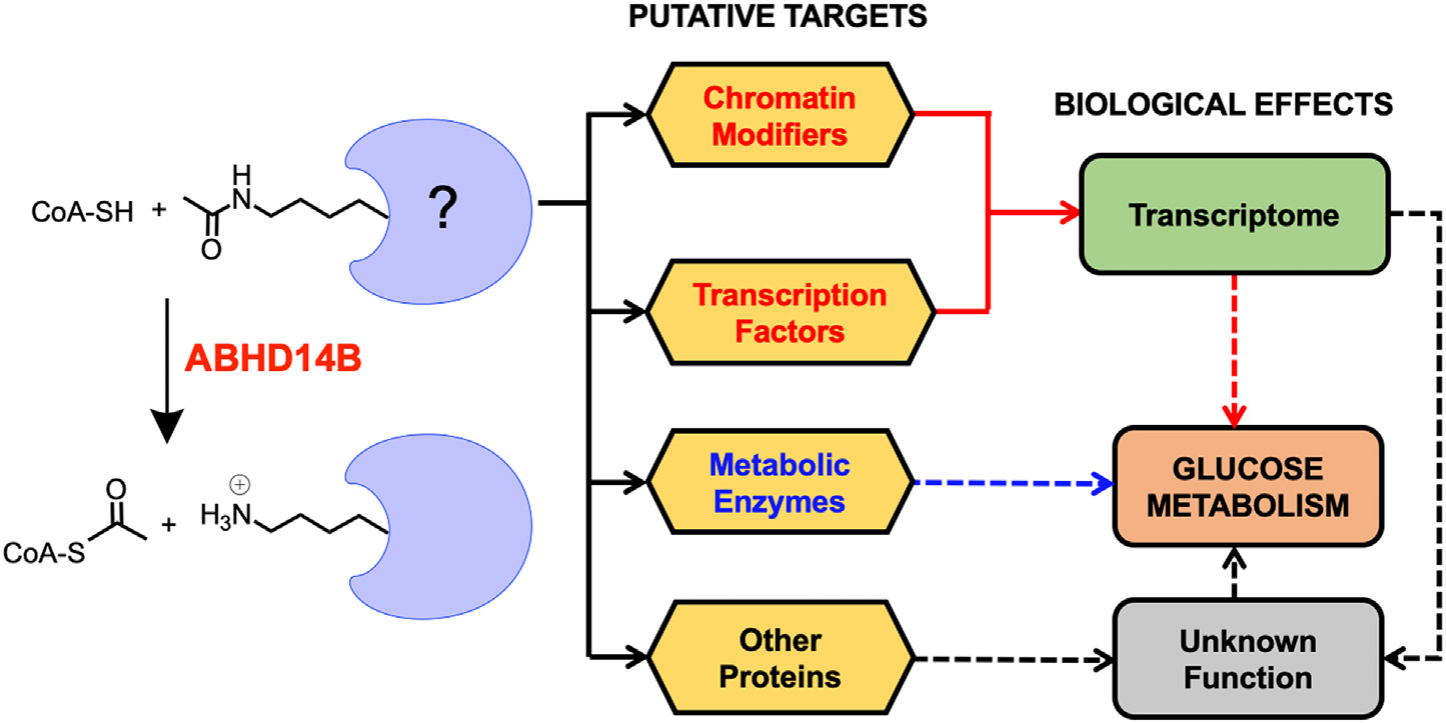
**Putative substrates of ABHD14B.** A schematic representation showing the possible functional proteins with PKAc that might be putative biological targets (substrates) of ABHD14B. These include: (i) chromatin modifiers and/or transcription factors that could produce changes at a transcript level to alter expression of key proteins involved in glucose metabolism; (ii) metabolic enzymes involved in glucose metabolic pathways, where PKAc is an important PTM for modulation of its activity; or (iii) unannotated proteins, whose role in glucose metabolism might yet not be known. ABHD14B, α/β-hydrolase domain containing protein # 14B; PKAc, protein lysine acetylation; PTM, posttranslational modification.

Projecting ahead, our findings open several research avenues for this interesting yet, cryptic metabolism regulating KDAC. First, all our cellular metabolite measurements performed were done at steady state conditions and to tease out further mechanistic details of the glucose metabolism (*e.g.,* lactate secretion, individual contributions of glycolysis and TCA cycle and gluconeogenesis, interplay glucose and lipid metabolism) regulated by ABHD14B, pulse-chase and isotopic-tracer based fluxomics experiments are definitely needed. Secondly, we have shown that ABHD14B plays an important role in regulating central glucose metabolism, and it would be interesting to study how depleted ABHD14B activity affects physiological processes intricately associated with glucose metabolism (*e.g.,* cell growth and division and mitochondrial respiration). Of note here, from immunoblotting experiments, we found that besides the liver and kidney, ABHD14B is also expressed in the pancreas (Fig. S3*D*), and it would be important to understand how through its KDAC activity, ABHD14B regulates the physiological interplay of glucose metabolism with hormonal levels of insulin/glucagon during feeding-fasting cycles. Thirdly, the endogenous substrates, *i.e.,* acetylated-lysine residues of proteins, are currently unknown, and an obvious follow-up study would be the identification of these substrates. The knowledge of the functional crosstalk of these substrates (*e.g.,* metabolic enzymes, transcriptional factors, chromatin modifiers, and proteins with as-of-yet unknown functions) associated with metabolism (Fig. 6), with ABHD14B in regulating glucose metabolism would be important in the years to come, especially in the context of human diseases. Emerging mass spectrometry–based chemoproteomics platforms have enabled the profiling of functional lysine residues in the proteome (64, 65), and these platforms can be leveraged to identify the posttranslationally modified acetylated-lysine residues of protein that are endogenous substrates for ABHD14B. Finally, an altered glucose metabolism can, in principle, be used as a physiological trigger in the treatment of central obesity (66) and inhibiting cancer tumor progression and survival (51). To test this hypothesis and ascertain the therapeutic role of ABHD14B in these physiological processes, the development of pharmacological tools (selective *in vivo* active inhibitors) and/or genetic models (knockout mice) for ABHD14B is much needed.

## Significance

In the post-genomic era, assigning functions to hitherto unannotated proteins particularly enzymes has greatly facilitated our understanding of the metabolic pathways that these regulate *in vivo*. We recently functionally annotated the orphan serine hydrolase enzyme ABHD14B as a KDAC, and following up on this discovery, we wanted to map the biological pathways that this cryptic enzyme regulates. Using complementary transcriptomics and metabolomics analysis, we show that depletion of ABHD14B in mammalian cells results in significantly altered glucose metabolism. Further, using a mouse model, we report a complex glucoregulatory phenotype modulated by hepatic ABHD14B and confirm the role of this novel KDAC in controlling glucose metabolism *in vivo*, especially while fasting.

## Experimental procedures

### Materials

Unless otherwise mentioned, media and consumables for mammalian cell cultures were purchased from HiMedia; chemicals, and reagents were purchased from Sigma-Aldrich; MS grade solvents were purchased from JT Baker; and chromatography columns and related accessories for LC-MS/MS analysis were purchased from Phenomenex.

### Mammalian cell culture

HEK293T cells were purchased from ATCC (catalog# CVCL_0063). Generation of a NT shRNA control line and two ABHD14B stable knockdown lines (KD_2 and KD_3) of HEK293T cells was previously reported by us (24). All cell lines were cultured in complete medium [RPMI1640 (HiMedia, catalog# AL028A) supplemented with 10% (v/v) fetal bovine serum (FBS, Brazil source {HiMedia, catalog# RM1112}) and 1% (v/v) penicillin-streptomycin (MP Biomedical, catalog# 1670049)] at 37 ^o^C and 5% (v/v) CO_2_. Additionally, the complete medium was supplemented with 6 μg/ml puromycin (MP Biomedical, catalog# 194539) for the NT and the ABHD14B knockdown (KD_2 and KD_3) HEK293T cells to specifically select only for the puromycin-resistant cells. All cells were cultured in 10 cm tissue culture dishes, and upon reaching 40 to 50% confluence, the spent media were replaced with a fresh complete medium containing 6 μg/ml puromycin. Upon 80 to 85% confluence, the cells were harvested by scraping, washed with cold Dulbecco’s phosphate-buffered saline (DPBS) (HiMedia, catalog# TL1006) (2-times), and centrifuged at 200*g* for 3 min to get the cell pellet. The harvested cell pellets were flash-frozen using liquid nitrogen and stored at −80 ^°^C until further use. All cell lines described here were routinely stained with 4′,6-diamidino-2-phenylindole (Sigma, catalog# D9542) and visualized by microscopy to ensure that they were devoid of any *mycoplasma* contamination using established protocols (24, 32, 67).

### RNA extraction, cDNA library synthesis, and sequencing

Total RNA was extracted from four biological replicates each of WT, NT, KD_2, and KD_3 HEK293T cells using QIAzol (Qiagen, catalog# 79306) and RNeasy Plus Universal Mini Kit (Qiagen, catalog# 73404) as per manufacturer’s instruction. RNA concentration was found out using a Nanodrop 2000c spectrophotometer (Thermo Fisher Scientific), and the cDNA library was synthesized from 300 ng of RNA using QuantSeq 3′ mRNA-Seq Library Prep Kit FWD for Illumina (Lexogen, catalog# 015.96) as per the manufacturer’s instruction. The concentration of the cDNA library was assessed using Qubit dsDNA HS kit (Invitrogen, catalog# Q32851) on a Qubit four Fluorimeter (Thermo Fisher Scientific). The average size of the library was assessed using a High Sensitivity DNA Kit (Agilent, catalog# 5067–4626) on a 2100 Bioanalyzer (Agilent). Equal amounts of the libraries were pooled, and 2 nmoles was sequenced using NextSeq 500/550 Mid Output Kit v2.5 (150 Cycles) (Illumina, catalog# 20024904) on NextSeq 550 instrument (Illumina). The read was single-ended and 76 bp long. The raw sequence data is deposited in the Gene Expression Omnibus (NCBI) repository (accession ID: GSE196907) and is publicly available.

### Transcriptome assembly and differential gene expression analysis

After trimming the adapters, the sequences were assessed for quality (FastQC) and mapped to human genome GRCh38 on the BlueBee Genomics Platform (Lexogen). The average depth of sequencing was 12 M, and a principal component analysis was performed on all samples using a custom R-script to determine the relatedness and clustering of the raw data. The differential gene expression analysis was performed on the Bluebee platform (Illumina) for NT, KD_2 and, KD_3 HEK293T cells with respect to WT HEK293T cells and visualized using volcano plots. After applying a false discovery rate threshold of an adjusted *p*-value cut-off of < 0.01 and a fold-change cut-off of > 1.5, the DEGs from NT, KD_2 and KD_3 HEK293T cells were assessed further using a Venn diagram created using Venny 2.1.0. (https://bioinfogp.cnb.csic.es/tools/venny/index.html). The DEGs in the KD_2 and KD_3 HEK293T cells overlapping with the NT HEK293T cells were removed from further bioinformatics analysis to negate the effect of off-targets coming from the NT shRNA plasmid. GO classification of the DEGs was initially performed on PANTHER classification system (29). An exhaustive list of DEGs involved in metabolism was also extracted using the MGI database (30), and a hierarchical clustering analysis of DEGs involved in metabolism from all samples was done using a custom R-script. Pathway enrichment analysis was also performed on the metabolic DEGs using the BINGO plugin for Cytoscape (31).

### Real-time qPCR analysis

The cDNA was extracted from 2 μg of RNA using the High-Capacity cDNA Reverse transcription kit (Thermo Fisher Scientific, catalog# 4374966) as per the manufacturer’s instructions. Real-time qPCR was performed on 1:5 or 1:3 diluted cDNA using gene-specific primers (see Table S1 for details of primers) with KAPA SYBR FAST Universal (Sigma-Aldrich, catalog# KK4602) in an Eppendorf Mastercycler RealPlex system.

### Metabolite extraction and LC-MS/MS analysis

Polar metabolites were extracted from cells using a reported protocol with minor technical modifications (34). Briefly, the cell pellets were resuspended in 600 μl of 75% (v/v) ethanol with respective internal standards [2 nmol of ^13^C-glucose (Cambridge Isotopes, catalog# CLM-1396) for non-derivatized metabolites and 0.1 nmol of D4-succinic acid (Cambridge Isotopes, catalog# DLM-2307) for derivatized metabolites]. The cell suspension was incubated at 80 ^°^C for 3 min with constant shaking and immediately kept on ice for 5 min. The mixture was then centrifuged at 20,000*g* for 10 min at 4 ^°^C, following which, the supernatant containing the desired polar metabolites was transferred to a new tube, dried under vacuum, and stored at −40 ^°^C until LC-MS/MS analysis. To study the TCA cycle intermediates, the dried extract was derivatized using a reported protocol (34). Briefly, the dried extract was resuspended in 150 μl of water and 75 μl of 1 M N-(3-dimethylaminopropyl)-N′-ethylcarbodiimide (prepared in 13.5 mM pyridine buffer, pH 5.0) (Sigma, catalog# E7750) and mixed gently. Thereafter, 150 μl of 0.5 M *O*-benzylhydroxylamine (Sigma, catalog# B22984), prepared in 13.5 mM pyridine buffer at pH 5.0, was added to the above mixture and mixed by shaking for 1 h at 25 ^°^C. Subsequently, 350 μl of ethyl acetate was added to the mixture and mixed by shaking for 10 min followed by centrifugation at 3,000*g* for 5 min at 4 ^°^C. The top layer was transferred in a new vial, and the ethyl acetate extraction was done two more times. The top layers from all three rounds of extraction were pooled together, dried under vacuum, and stored at −40 ^°^C until LC-MS/MS analysis. The amino acids from cells were extracted using an established protocol (35). Briefly, cell pellets were resuspended in 200 μl of 80% (v/v) methanol containing 2 nmol ^13^C-alanine (Cambridge Isotopes, catalog# CLM-116) as an internal standard, vortexed, and incubated on ice for 10 min. The mixture was then centrifuged at 15,000*g* for 10 min at 4^o^C. For every 70 μl of the supernatant, 30 μl of 1.7 mM perfluoroheptanoic acid or tridecafluoroheptanoic acid was added and mixed. The metabolites were stored at −40 ^°^C until the LC-MS/MS analysis. The extraction of CoA and esters of CoA from cells was done using a protocol described earlier by us (24). All lipids from cell pellets were extracted and analyzed using LC-MS/MS using an established protocol described by us (33, 68). Internal standards used for lipid measurements were 1 nmol heptadecenoic acid (Sigma, catalog# H8896) for negative ion mode and 50 pmol of C17:0/20:4 phosphatidylcholine (Avanti Polar Lipids, catalog# LM-1002) for positive ion mode for relative quantification of lipids. All the samples were analyzed on a Sciex X500R quadrupole time-of-flight mass spectrometer fitted with an Exion UHPLC system (Sciex). The dried metabolites were resuspended in appropriate solvent using a bath sonicator and subsequently centrifuged at 20,000*g* for 5 min at 4 ^°^C. The supernatant was injected onto either a Phenomenex Gemini C18 column (50 mm × 4.6 mm, 5 μm, 110 Å) (catalog# 00B-4435-E0), a Phenomenex Luna C5 column (50 mm × 4.6 mm, 5 μm, 110 Å) (catalog# 00B-4043-E0), or a Phenomenex Synergi Fusion-RP Column (150 mm x 4.6 mm, 4 μm, 80 Å) (catalog# 00F-4424-E0) fitted with a Phenomenex guard column (3.2 mm X 8.0 mm) (catalog# KJ0-4282) using the Exion UHPLC system. Derivatized, nonderivatized polar metabolites and esters of CoA were quantified using data-independent acquisition, whereas amino acids and nonpolar metabolites were quantified using the multiple reaction monitoring method. All details of the LC profiles and MS parameters are provided in Table S2. All the data were collected and analyzed using the SCIEX OS software. All the metabolites were quantified by measuring the area under the curve, compared to that of the respective internal standard, and then normalized to the total protein content of the respective cell pellet. Additionally, to negate any off-target effects of the NT HEK293T cells, all experimental groups, namely KD_2, KD_3, and NT HEK293T cells, were further normalized to WT HEK293T cells to get normalized relative values of metabolites for further analysis.

### Lactate production assay

All the HEK293T cell lines were grown in 6-well plates in complete RPMI1640 medium described earlier. Upon reaching 80% confluence, the media were removed, the cells were washed with sterile DPBS (3-times), and the media was replaced with sterile DPBS containing 10% (v/v) FBS, 2 mg/ml glucose. The spent media were collected at 0 and 6 h after this media change, following which the cells were harvested. The levels of lactate in the spent media and cells were measured using the LC-MS method described above and normalized to the protein concentration of the harvested cells. The cellular and secreted levels of lactate from KD_2 and KD_3 HEK293T cells were normalized to the corresponding levels of WT (or NT) HEK293T cells and are reported as fold changes relative to WT HEK293T cells.

### Liver-specific knockdown in mice

All studies were conducted in 10- to 12-week-old C57BL/6J mice. Mice were housed in the National Facility for Gene Function in Health and Disease at IISER Pune. All animal experiments described here have received formal approval from the Institutional Animal Ethics Committee, IISER Pune (IAEC-IISER Pune) as per guidelines provided by the Committee for the Purpose of Control and Supervision of Experiments on Animals constituted by the Government of India (No: IISER_Pune IAEC/2019_2/08). The experimental animals were housed in the National Facility for Gene Function in Health and Disease as per IAEC-IISER Pune policy on the social housing of animals. The hepatic knockdown of ABHD14B was achieved using the *In vivo*-jetPEI-Gal transfection reagent (Polyplus Transfection, catalog# 202–10G, updated catalog # 101000047) as per the manufacturer’s instructions using shRNA coding plasmids targeting ABHD14B reported earlier (24). For measuring fasting blood glucose levels, mice were not given food for 6 h but had *ad libitum* access to water. The GTT and PTT in mice were performed as per established protocols (36).

### ELISA and Western blot experiments

The hepatic ATP (catalog # ab83355), serum insulin (catalog # ab278123), and serum glucagon (catalog # ab267567) levels were measured using commercially available ELISA kits from Abcam as per the manufacturer’s protocol. All Western blot experiments on cell lysates and tissues were done using established protocols described by us (24, 69, 70). All blots were developed using the SuperSignal West Pico PLUS Chemiluminescent substrate (Thermo Fisher Scientific #34580) and imaged thereafter on a Syngene Chemi-XRQ gel documentation system. The primary rabbit polyclonal anti-ABHD14B was developed and characterized in-house (24). Primary anti-GAPDH (catalog# ab8245) and anti-β-actin (catalog# ab8224) antibodies were purchased from Abcam, and the primary anti-α-Tubulin (catalog# T9026) antibody was purchased from Sigma-Aldrich. The secondary antibodies, anti-rabbit IgG HRP was purchased from Thermo Fisher Scientific (catalog# 31,460), and anti-mouse IgG HRP was purchased from Abcam (catalog# ab6789). All primary and secondary antibodies were used at a dilution of 1:1000 and 1:10,000, respectively.

### Quantitation and statistical analysis

Unless otherwise mentioned, all data presented in this paper are mean ± standard deviation for the biological replicates from independent experiments as reported for that experiment. Unless otherwise mentioned, all graphs, plots, and statistical analyses reported in this paper were made using the GraphPad Prism 9 (version 9.3.1 (350)) for Mac OS-X software. An unpaired Student’s *t* test was used to determine statistical significance between two groups, and a *p*-value < 0.05 was considered statistically significant in this study unless mentioned otherwise.

## Data availabilty

The raw sequence data for the transcriptomics studies in this study is deposited in the Gene Expression Omnibus (NCBI) repository (accession ID: GSE196907) and is publicly available. All the other data that support the findings of this study are available in the paper, and its associated supporting information, or is available from the corresponding author upon reasonable request.

## Supporting information

This article contains supporting information. Tables S1 and S2 (XLSX); Figs. S1–S3 (PDF)

## Conflict of interest

The authors declare that they have no conflicts of interest with the contents of this article.

## Supporting information

Rajendranetal_SI figures_r2_TC
