# Supplementary material for: A multi-omics analysis reveals that the lysine deacetylase ABHD14B influences glucose metabolism in mammals
Source: J Biol Chem. 2022 Jun 11;298(7):102128. doi: 10.1016/j.jbc.2022.102128 (PMC9270251; doi:10.1016/j.jbc.2022.102128)
Supplement: Rajendranetal_SI figures_r2_TC [file mmc1.pdf]

A multi-omics analysis reveals that the lysine deacetylase ABHD14B influences glucose metabolism in mammals

Abinaya Rajendran[1], Amarendranath Soory[1,2], Neha Khandelwal[1,3], Girish Ratnaparkhi[1], Siddhesh S. Kamat[1,*]

[1]Department of Biology, Indian Institute of Science Education and Research (IISER) Pune, Maharashtra, India 411008.

[2]Present Address: Babraham Institute, Cambridge CB22 3AT, United Kingdom.

[3]Present Address: Center for Cancer Research, Massachusetts General Hospital, 149 13th Street, Boston, MA 02129, USA.

*To whom the correspondence should be addressed: siddhesh@iiserpune.ac.in

SUPPORTING FIGURES

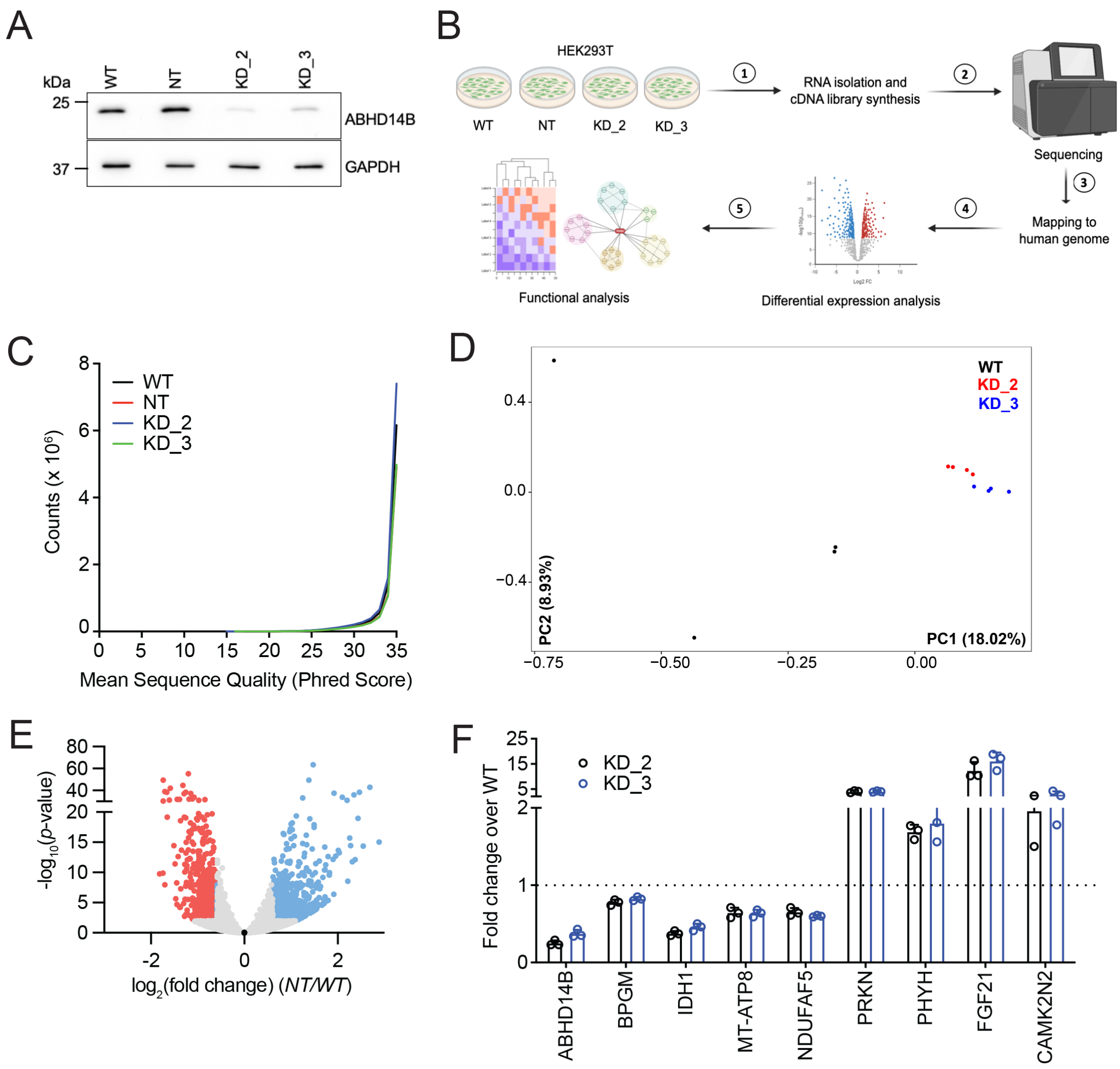

Figure S1. (**A**) Representative western blots confirming the knockdown of ABHD14B at a protein level (> 90%) in HEK293T cells following transfections with KD_2 or KD_3 plasmids, relative to a non-targeting (NT) plasmid or no plasmid WT HEK293T cells. GAPDH was used as protein loading control for this experiment. This western blot analysis was performed four times with reproducible results each time. (**B**) A schematic representation of the pipeline used for the RNA-sequencing experiments and downstream analysis of the transcriptomics data from HEK293T cells, where ABHD14B was depleted. (**C**) Plot showing the quality of the raw reads from the RNA-sequencing experiment, as depicted by "Counts" on the y-axis, and the "Mean Sequence Quality (Phred Score)" on the x-axis. Data represents mean from four biological replicates per experimental group. (**D**) A principal component analysis (PCA) for the gene expression data from various samples, showing tight clustering of the various experimental groups. Here each point represents a biological replicate. (**E**) Volcano plot showing differentially expressed genes (DEGs) in NT HEK293T cells relative to WT HEK293T cells as determined by RNA-sequencing. The data represents mean values from

four independent biological experiments. A cut-off of adjusted *p*-value < 0.01 was set for the genes, with a change of > 1.5-fold. Based on this filter, up-regulated and down-regulated genes are coloured in blue and red respectively. (**F**) qPCR analysis of selected DEGs from KD_2 and KD_3 HEK293T cells to validate findings from the transcriptomics experiments. All the raw data was first normalized to two housekeeping genes (actin or HPRT), and then values were plotted relative to levels observed in WT HEK293T cells. The data is represented as mean ± standard deviation from three independent biological experiments.

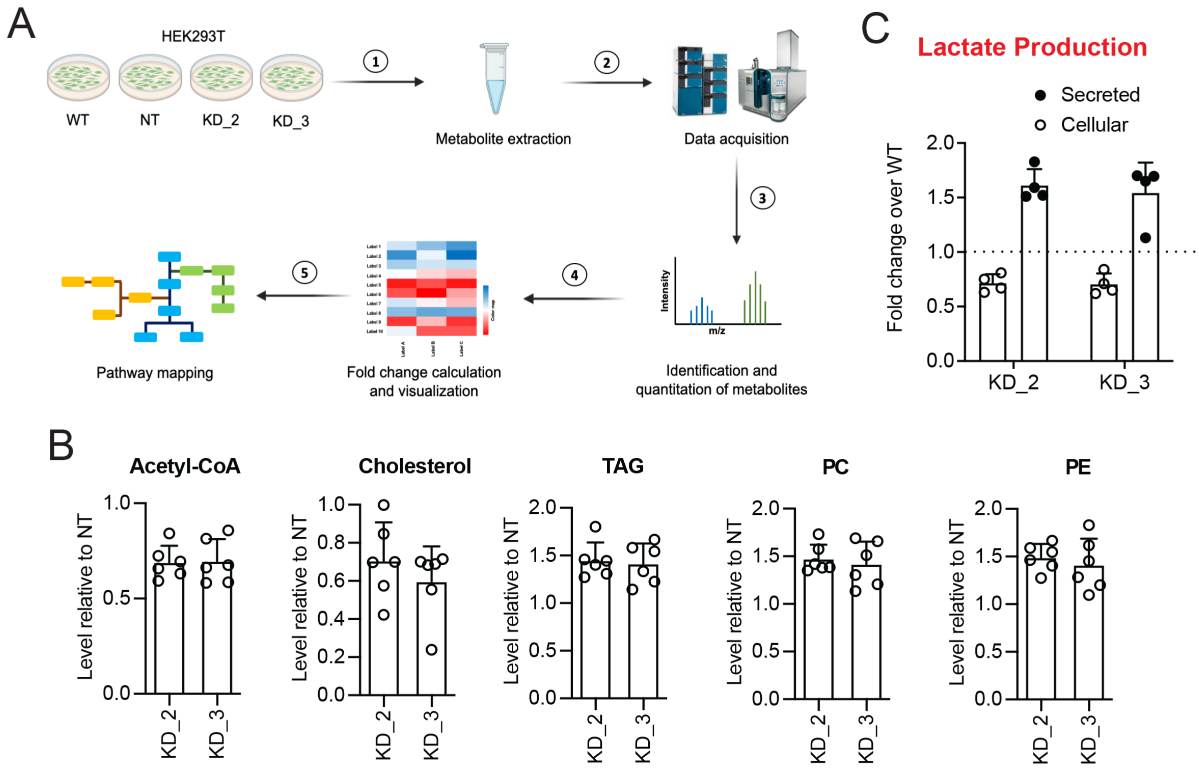

Figure S2. (**A**) A schematic representation of the pipeline employed for the LC-MS/MS based metabolomics experiments and downstream analysis of this data from HEK293T cells, where ABHD14B was knocked down. (**B**) Relative quantification of various metabolites (acetyl-CoA, cholesterol, triglycerides (TAG), phosphatidylcholine (PC), phosphatidylethanolamine (PE)) showing altered cellular concentrations in KD_2 and KD_3 HEK293T cells relative to NT HEK293T cells (all values normalized to WT HEK293T cells). For TAG, PC, and PE a weighted average (based on relative concentrations) for fold changes of all the species for that lipid class was calculated. Bars represents mean ± standard deviation from six biological replicates (independent experiments) per group. (C) A lactate production assay showing the decreased cellular and increased secreted levels of lactate from KD_2 and KD_3 HEK293T cells relative to WT (or NT) HEK293T cells. Bars represents mean ± standard deviation from four biological replicates (independent experiments) per group.

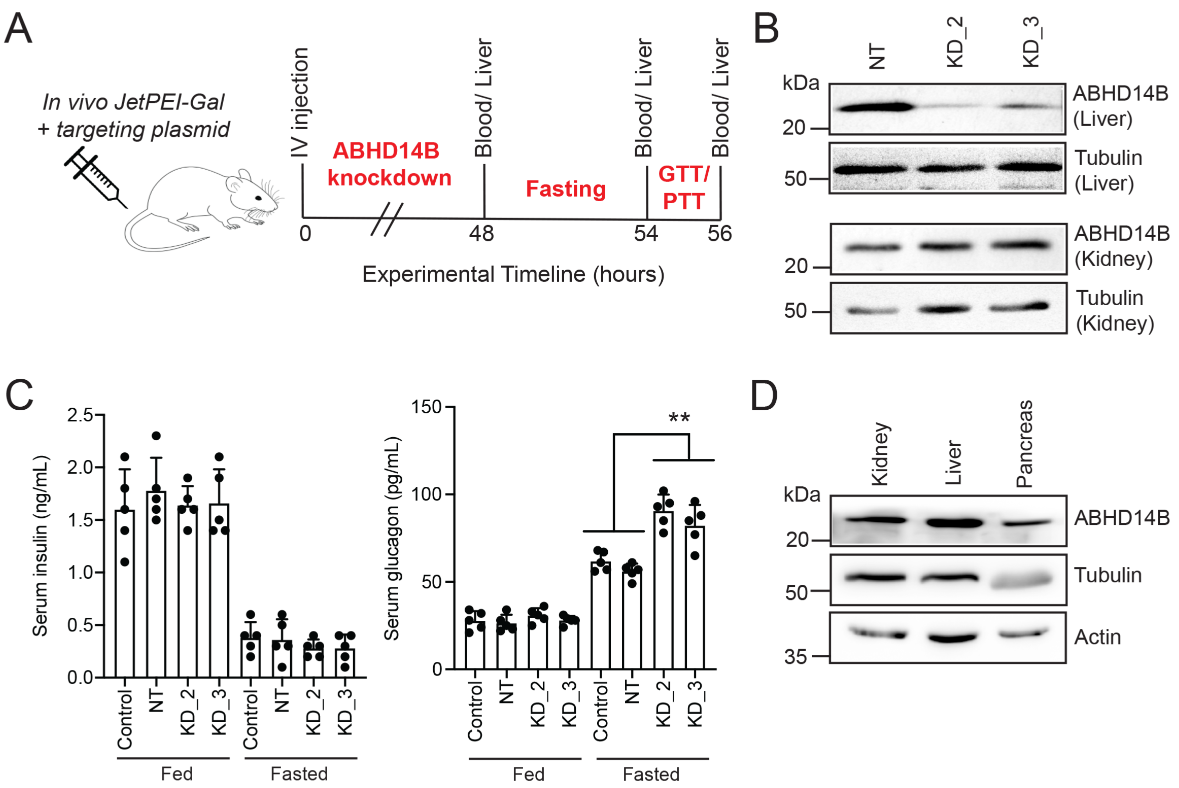

Figure S3. (**A**) A schematic representation of the experimental plan to study the *in vivo* effects of knocking down hepatic ABHD14B in mice using an established transfection strategy. (**B**) Representative western blots confirming the knockdown of ABHD14B at a protein level (> 90%) in mice liver, but no effect on ABHD14B at a protein level in kidneys of mice, following transfections with KD_2 or KD_3 plasmids, relative to a non-targeting (NT) plasmid 56 hours post-transfection at the end of the experiment as per the experimental plan represented in part (**A**). α-Tubulin was used as protein loading control for this immunoblotting experiment. This western blot analysis was performed three times with reproducible results each time. (**C**) Concentrations of serum insulin and glucagon in mice from different experimental groups during a fed and 6-hour fasted state. The data is represented as mean $\pm$ standard deviation of five biological replicates (independent experiments) per experimental group. Student's *t*-test (two tailed): **$p < 0.01$ KD_2 or KD_3 versus control (or NT) group. (**D**) Representative western blots confirming the expression of ABHD14B in the pancreas, along with the liver and kidneys. α-Tubulin and β-Actin were used as protein loading control for this immunoblotting experiment, where 25 μg of soluble lysate was used per tissue sample. This western blot analysis was performed three times with reproducible results each time.
